# Is the Newly Defined R_2_CHA_2_DS_2_-Vasc Score a Predictor for Late Mortality in Patients Undergoing Transcatheter Aortic Valve Replacement?

**DOI:** 10.21470/1678-9741-2019-0221

**Published:** 2020

**Authors:** Muhsin Kalyoncuoglu, Semi Ozturk

**Affiliations:** 1 Department of Cardiology, Haseki Training and Reseach Hospital, University of Health Sciences, Istanbul, Turkey.

**Keywords:** Aortic Valve, Transcatheter Aortic Valve Replacement, Confidence Intervals, Follow-Up Studies, Regression Analysis, Sensitivity ans Specificity, Algorithms

## Abstract

**Objective:**

To assess the performance of the modified R_2_CHA_2_DS_2_-VASc score for predicting mid-to-long-term mortality (> 30 days) in patients undergoing transcatheter aortic valve replacement (TAVR).

**Methods:**

Data of 78 patients who underwent TAVR were retrospectively reviewed. R_2_CHA_2_DS_2_-VASc score was compared with the European System for Cardiac Operative Risk Evaluation II (EuroSCORE II or ES II) and the transcatheter valve therapytranscatheter aortic valve replacement (TVT-TAVR) risk score.

**Results:**

The mean follow-up period was 17.4±9.9 months (maximum 37 months). Early mortality (first 30 days) was observed in 10 (12.8%) patients, whereas mid-to-long-term mortality (> 30 days) was observed in 26 (33.3%) patients. Non-survivors had higher values of R_2_CHA_2_DS_2_-VASc, ES II, and TAVR scores than survivors (*P*<0.001, *P*<0.001, and *P*=0.001, respectively). Analysis of Pearson’s correlation revealed that R_2_CHA_2_DS_2_-VASc score was moderately correlated with ES II and TAVR scores (*r*=0.51, *P*<0.001; *r*=0.44, *P*=0.001, respectively). Pairwise comparisons of R_2_CHA_2_DS_2_-VASc (area under the curve [AUC]: 0.870, 95% confidence interval [CI]: 0.776-0.964; *P*<0.001), ES II (AUC: 0.801, 95% CI: 0.703-0.899; *P*<0.001), and TAVR scores (AUC: 0.730, 95% CI: 0.610-852; *P*=0.002) showed similar accuracy for predicting mortality. R_2_CHA_2_DS_2_-VASc score is an independent predictor of mortality in multivariable Cox regression analysis. A cutoff value of six for R_2_CHA_2_DS_2_-VASc score showed a sensitivity of 74% and a specificity of 89% for predicting mid-to-long-term mortality.

**Conclusion:**

R_2_CHA_2_DS_2_-VASc score, easily calculated from clinical parameters, is associated with prediction of mid-to-longterm mortality in patients undergoing TAVR.

**Table:** 

Abbreviations, acronyms & symbols				
ACC	= American College of Cardiology		LBBB	= Left bundle branch block
AS	= Aortic stenosis		LES	= Logistic European System for Cardiac Operative Risk Evaluation
AUC	= Area under the curve		LVEF	= Left ventricular ejection fraction
AVA	= Aortic valve area		NYHA	= New York Heart Association
CAD	= Coronary artery disease		OR	= Odds ratio
CI	= Confidence interval		PAD	= Peripheral arterial disease
COPD	= Chronic obstructive pulmonary disease		PROM	= Predicted Risk of Mortality
CVA	= Cerebrovascular accident		RBBB	= Right bundle branch block
DM	= Diabetes mellitus		ROC	= Receiver operating characteristic
ECG	= Electrocardiogram		SAVR	= Surgical aortic valve replacement
eGFR	= Estimated glomerular filtration rate		sPAP	= Pulmonary artery systolic pressure
EuroSCORE II	= European System for Cardiac Operative Risk Evaluation II		STS	= Society of Thoracic Surgeons
			TAVI	= Transcatheter aortic valve implantation
ES II	= European System for Cardiac Operative Risk Evaluation II		TAVR	= Transcatheter aortic valve replacement
FBG	= Fasting blood glucose		TIA	= Transient ischemic attack
HF	= Heart failure		TVT	= Transcatheter valve therapy
HT	= Hypertension		VARC	= Valve Academic Research Consortium
IS	= Ischemic stroke		WBC	= White blood cells

## INTRODUCTION

Transcatheter aortic valve replacement (TAVR) has emerged as an alternative to surgical aortic valve replacement (SAVR) for the treatment of patients with symptomatic severe aortic stenosis (AS) who are not candidates for surgery or those considered to be at high risk for adverse postsurgical outcomes^[1,2]^. In the absence of an established TAVR-specific scoring system to stratify patients undergoing TAVR, the Society of Thoracic Surgeons (STS) Predicted Risk of Mortality (PROM) score, the Logistic European System for Cardiac Operative Risk Evaluation (LES) score, and the European System for Cardiac Operative Risk Evaluation II (EuroSCORE II) are most commonly integrated in the heart team evaluation of patients with symptomatic severe AS in order to predict their risk of mortality^[3]^. However, these options are mainly used in clinical practice to estimate surgical risk prior to SAVR and were developed based on a patient population that is different from the typical cohort of elderly transcatheter aortic valve implantation (TAVI) patients with comorbidities. As such, their applicability among patients with TAVR is still controversial. Thus, several new risk models specific to TAVR have been recently developed, such as the STS/American College of Cardiology (ACC) transcatheter valve therapy (TVT)-TAVR risk score (TAVR score)^[4]^. However, many of these scores have not been validated in external cohorts, which have limited their adoption in clinical practice^[5-8]^. Another score, the CHA_2_DS_2_-VASc, is a well-validated option to establish the risk of cerebrovascular accident (CVA) in patients with nonvalvular atrial fibrillation^[9]^. Recently, it was also proposed that the CHA_2_DS_2_-VASc score and its modified version (R_2_CHA_2_DS_2_-VASc score) are able to discern 30-day mortality in patients undergoing TAVR^[10]^. To the best of our knowledge, no data exist evaluating the predictive value of the newly defined R_2_CHA_2_DS_2_-VASc score for late mortality in patients undergoing TAVR. The present study therefore aimed to assess the performance of the modified R_2_CHA_2_DS_2_-VASc score for predicting mid-to-long-term mortality (> 30 days) in patients undergoing TAVR.

## METHODS

Eighty consecutive patients who underwent TAVR were retrospectively analyzed and their data, which were collected as part of routine clinical practice at Haseki Training and Research Hospital between January 2015 and March 2018, were reviewed. These data, including clinical assessment findings, electrocardiogram (ECG), chest X-ray, echocardiogram, multislice computed tomography of the aorta and branches, cine coronary angiography, and laboratory test results, were obtained from a computerised system and/or patient file records or during follow-up visits.

Severe AS is defined as a valvular orifice area < 1.0 cm^2^ or < 0.6 cm^2^/m^2^ and/or a mean pressure gradient > 40 mmHg and/ or a jet velocity > 4.0 m/s. The exclusion criteria of the present study were: patients undergoing combined procedures, such as concurrent percutaneous coronary intervention, patients with an estimated life expectancy of less than one year, patients with a significant mental impairment, and patients with insufficient data. Of the 80 patients, one (1.3%) with insufficient information and one who underwent simultaneous percutaneous coronary intervention and TAVR were excluded. Consequently, the study population included 78 patients with a life expectancy of at least one year who were considered to be at high risk for surgical complications on the basis of clinical assessments performed by a multidisciplinary heart team^[2,3]^. The heart team used a guideline that was based on a risk model developed by the EuroSCORE II to estimate the risk of death after index TAVR procedure. EuroSCORE II and TAVR score were calculated using online tools (www.euroscore.org and https://tools.acc.org/tavrrisk, respectively).

Chronic heart failure (HF) was defined as a history of HF signs and symptoms confirmed with objective evidence of cardiac dysfunction or reduced left ventricular ejection fraction (LVEF) (< 40%). Type 2 diabetes mellitus (DM) was defined as a previous diagnosis and/or fasting glucose > 125 mg/dL or treatment with oral hypoglycemic agent and/or insulin. Hypertension (HT) was defined as a resting blood pressure > 140/90 mmHg on at least two occasions or current antihypertensive pharmacologic treatment. Vascular disease was defined as a history of prior myocardial infarction and/or peripheral arterial disease (PAD) including prior revascularisation. PAD was defined as atherosclerotic disease in the aorta and arteries other than the coronaries, with exercise-related claudication, revascularization therapy, reduced or absent pulsation, or amputation or angiographic stenosis > 50%. Ischemic stroke (IS) and transient ischemic attack (TIA) were evaluated according to the history given by the patients.

Outcomes (*e.g*., overall mortality, cardiovascular mortality, pacemaker requirement, overall bleeding, major bleeding, CVA, major vascular complications, and overall vascular complications) were adjudicated according to the Valve Academic Research Consortium (VARC)-2 criteria^[11]^. The prespecified primary endpoint of the trial was all-cause mortality within 30 days (defined as early mortality) and that at more than 30 days (defined as mid-to-long-term or late mortality) for the study cohort. The follow-up adherence rate was 100% among all patients after the index operation.

The default access option for TAVR was transfemoral and valve selection was performed at the discretion of the heart team. The procedure was completed in the cardiac catheterization laboratory under general anesthesia or sedoanalgesia with transesophageal echocardiography guidance. All patients received aspirin (81 mg) and clopidogrel (≥ 300 mg) before the procedure and heparin during the procedure; patients continued to take aspirin indefinitely and clopidogrel for a minimum of one month postoperation. After the index procedure, all patients were followed up for 30 days, six and 12 months, and yearly thereafter. Since our study was retrospectively designed, written informed consent from the participants could not be obtained, but our study protocol conformed to the principles of the Declaration of Helsinki and was approved by the local ethics committee of our institution.

### R_2_CHA_2_DS_2_-Vasc Score Calculation

The meaning of R_2_CHA_2_DS_2_-VASc can be explained as follows: R_2_ relates to preexisting renal impairment (serum creatinine > 200 µmol/L or estimated glomerular filtration rate [eGFR] < 60 mL/min/1.73 m^2^) and/or to preexisting conduction abnormality such as right bundle branch block (RBBB) or left bundle branch block (LBBB) on preprocedural ECG, while C refers to congestive HF, H refers to HT, A_2_ refers to an age of 75 years or more, D refers to DM, S_2_ refers to a history of IS or TIA, V refers to vascular disease, A refers to an age of between 65 and 74 years, and Sc refers to female sex. R_2_CHA_2_DS_2_-VASc scores were calculated for all patients by assigning one point for each of the following criteria: renal impairment, conduction abnormality, age of 65 to 75 years, HT, DM, HF, female sex, and vascular disease and two points each for a history of IS or TIA and an age of 75 years or more. The maximum R_2_CHA_2_DS_2_-VASc score possible was 11 points (Table 1).

**Table 1:** Definition of R_2_CHA_2_DS_2_-VASc score.

Nomenclature	R_2_CHA_2_DS_2_-VASc	Point
R	Renal impairment	1 point
R	RBBB/LBBB on 12-lead ECG	1 point
C	Congestive heart failure	1 point
H	Hypertension	1 point
A_2_	Age > 75 years	2 points
D	Diabetes mellitus	1 point
S_2_	Previous stroke or TIA	2 points
V	Vascular disease	1 point
A	Age 65-74 years	1 point
Sc	Sex category, female gender	1 point

ECG=electrocardiogram; LBBB=left bundle branch block; RBBB=right bundle branch block; TIA=transient ischemic attack

### Statistical Analysis

Statistical analyses were performed using the Statistical Package for the Social Sciences software (IBM Corp., Armonk, NY, USA), version 24.0. Continuous variables are given as means ± standard deviations if normally distributed or medians (interquartile ranges) if not normally distributed. The categorical variables are given as percentages. Chi-squared (χ^2^) test was used to compare the categorical variables among the groups. Kolmogorov-Smirnov test was used to assess whether the variables were normally distributed or not. Student’s *t*-test or Mann-Whitney U test was used to compare the continuous variables between the groups according to whether they were normally distributed or not. To identify predictors of early and mid-to-long-term mortality, univariable and multivariable Cox proportional hazards regression analyses were performed. For all regression analyses, only variables with a *P*-value < 0.1 in a univariable analysis were incorporated in the multivariable model. To avoid model overfitting, EuroSCORE II and TAVR score were not included in the same multivariable regression model. Furthermore, variables already considered by the R_2_CHA_2_DS_2_-VASc score, EuroSCORE II, or TAVR score were not evaluated separately in any multivariable analysis independently of their significance in a univariable analysis. Discriminatory power and identification of the sensitivity and specificity of R_2_CHA_2_DS_2_-VASc, ES II, and TAVR scores for mid-to-long-term mortality were assesed by calculating the C-statistic (the area under the receiver operating curve). The optimal cutoff value for the R_2_CHA_2_DS_2_-VASc score was calculated from the point of maximal sensitivity and specificity (Youden’s index). To compare the predictive performance of those scores, pairwise comparison of receiver operating characteristic (ROC) curves by using DeLong et al.^[12]^ was also analysed. Correlations between R_2_CHA_2_DS_2_-VASc, EuroSCORE II, and TAVR scores were depicted in scatterplot diagrams. Pearsons’s correlation coefficient was calculated to describe the degree of correlation. Kaplan-Meier survival curves were used to depict the early and mid-to-long-term survival patterns of patients who were stratified into high- and low-risk groups by using a R_2_CHA_2_DS_2_-VASc score cutoff of six points. The results were evaluated within 95% confidence interval (CI) and at a significance level of *P*<0.05.

## RESULTS

### Baseline Characteristics

The mean follow-up period was 17.4±9.9 months, with a maximum length of 37 months. The mean age was 76.3±8.4 years and 47 (60.3%) patients were female. Admission diagnosis was HF in 53 (67.9%) patients, angina or angina-equivalent symptoms in 20 (25.6%) patients, and presyncope or syncope in five (6.4%) patients, with no individuals suffering sudden cardiac death. Mean aortic valve area (AVA) was 0.61±0.12 cm^2^ and mean transvalvular gradient was 50.6±6.1 mmHg. Twenty-three (29.5%) patients had severe symptoms defined as New York Heart Association (NYHA) functional classes III to IV. The mean R_2_CHA_2_DS_2_-VASc score was 4.8±1.7 points, mean EuroSCORE II was 5.2±2.1 (%), and mean TAVR score was 3.7±1.7 (%). Detailed demographic, clinical, and echocardiographic characteristics of the study population are summarised in Table 2. The procedure was performed by using conscious sedation in 64 (82.1%) patients and general anesthesia in 14 (17.9%) patients. A total of 48 (61.5%) patients received CoreValve™ (Medtronic Inc., Minneapolis, MN, USA), while 27 (34.6%) patients received Edwards-SAPIEN™ (Edwards Lifesciences, Irvine, CA, USA), and three (3.8%) patients received Portico™ (Abbott Laboratories, Chicago, IL, USA). Major VARC-2-defined procedure-related complications occurred in 24 (29.6%) patients. Complications included new pacemaker insertion in 13 (16.7%) patients, any VARC-2-defined major vascular injury in 11 (14.1%) patients, major bleeding in 14 (17.9%) patients, and acute renal failure in 10 (12.8%) patients. Three (3.7%) patients required dialysis treatment. None of the study participants experienced a permanent stroke or needed surgical intervention either peri-TAVI or post-TAVI. Periprocedural characteristics of the study population are summarised in Table 3.

**Table 2:** Demographic, clinical, and laboratory parameters of study population before the index procedure.

Variables	All population	Alive n=52 (66.7%)	Early dead n=10 (12.8%)	*P*	Late dead n=26 (33.3%)	*P*
Male gender, n %	47 (60.3)	33 (63.5)	6 (60)	0.9	14 (53.8)	0.4
Age	76.3±8.4	75.3±9.0	79.2±6.9	0.2	78.9±6.0	0.1
Hypertension, n (%)	50 (64.1)	38 (69.1)	6 (60)	0.7	12 (52.2)	0.16
Diabetes mellitus, n (%)	25 (32.1)	14 (25.5)	7 (70)	0.006	11 (47.8)	0.05
Heart failure, n (%)	37 (47.4)	20 (36.4)	9 (90)	0.004	17 (73.9)	0.002
Vascular disease history, n (%)	33 (42.3)	19 (34.5)	4 (40)	0.9	14 (60.9)	0.03
CVA history, n (%)	7 (9)	2 (3.6)	2 (20)	0.19	5 (21.7)	0.01
COPD, n (%)	25 (32.1)	16 (29.1)	6 (60)	0.04	9 (39.1)	0.38
NYHA class III-IV, n (%)	23 (29.5)	10 (18.2)	7 (70)	0.003	13 (56.5)	0.001
Atrial fibrillation, n (%)	16 (20.5)	9 (16.4)	3 (30)	0.4	7 (30.4)	0.16
Presence of RBBB or LBBB, n (%)	17 (21.8)	7 (12.7)	4 (40)	0.13	10 (43.5)	0.003
Aortic valve area, cm^2^	0.61±0.12	0.62±0.13	0.54±0.07	0.043	0.6±0.11	0.5
Mean aortic valve gradient, mmHg	50.6±6.1	50.4±6.3	54.2±3.7	0.046	51±5.6	0.76
Left ventricular ejection fraction, %	47.8±8.3	50.9±5.6	38±5.4	<0.001	41.3±5.3	<0.001
sPAP, mmHg	49±9.8	46.6±8.6	58.7±7.6	<0.001	52.9±8	0.004
FBG, mg/Dl	141±63.2	134.2±52.5	167±67	0.17	157.2±82.7	0.15
eGFR, mL/min	66.7±24.4	71.8±22.9	42.2±24.9	<0.001	54.6±23.9	0.004
Hematocrit, %	35.5±4.5	36.1±4.4	36.8±4.6	0.34	34.4±4.6	0.12
WBC, 10^3^/µL	8.1±3.7	7.9±3.8	8.1±2.2	0.17	8.1±3.3	0.4
Platelet, 10^3^/µL	230.7±75.3	222.3±68.1	206.4±78	0.28	237.5±89	0.6
CHA_2_DS_2_-VASc score	4.3±1.3	3.82±0.9	5.6±0.8	<0.001	5.3±1.2	<0.001
R_2_CHA_2_DS_2_-VASc score	4.8±1.7	4.2±1.3	6.8±1.3	<0.001	6.4±1.4	<0.001
EuroSCORE II, %	5.2±2.1	4.7±1.8	7.9±1.7	<0.001	6.6±1.9	<0.001
TAVR score, %	3.7±1.7	3.3±1.5	5.8±1.6	<0.001	4.7±1.8	0.001

COPD=chronic obstructive pulmonary disease; CVA=cerebrovascular accident; eGFR=estimated glomerular filtration rate; EuroSCORE II=European System for Cardiac Operative Risk Evaluation II; FBG=fasting blood glucose; LBBB=left bundle branch block; NYHA=New York Heart Association; RBBB=right bundle branch block; sPAP=pulmonary artery systolic pressure; TAVR=transcatheter aortic valve replacement; WBC=white blood cells

**Table 3:** Procedural and postprocedural parameters of study population during the follow-up period.

Variables	All population	Alive n=52 (66.7%)	Early dead n=10 (12.8%)	*P*	Late dead n=26 (33.3%)	*P*
Conscious sedation, n %	64 (82,1)	44 (80)	7 (70)	0.28	20 (87)	0.47
Type of valve, n(%)				0.4		0.49
CoreValve™	48 (61.5)	34 (61.8)	8 (80)		14 (60.9)	
Edwards-SAPIEN™	27 (34.6)	18 (32.7)	2 (20)		9 (39.1)	
Portıco™	3 (3.8)	3 (5.5)	0 (0)		0 (0)	
Predilatation, n (%)	36 (46.2)	25 (45.5)	2 (20)	0.08	11 (47.8)	0.8
Postdilatation, n (%)	14 (17.9)	11 (20)	1 (10)	0.49	3 (13)	0.5
Implantation depth, mm	5.3±0.7	5.3±0.7	5.4±0.6	0.6	5.2±0.8	0.8
Paravalvular leakage (> 2+), n (%)	8 (10.3)	4 (7.3)	2 (20)	0.28	4 (17.4)	0.18
Major vascular complications, n (%)	11 (14.1)	9 (16.4)	1 (10)	0.69	2 (8.7)	0.38
Bleeding complications, n (%)	14 (17.9)	7 (12.7)	5 (50)	0.005	7 (30.4)	0.06
Pericardial tamponade, n (%)	8 (10.3)	3 (5.5)	4 (40)	0.001	5 (21.7)	0.03
Acute renal failure, n (%)	10 (12.8)	3 (5.5)	5 (50)	<0.001	7 (30.4)	0.003
Permanent pacemaker, n (%)	13 (16.7)	9 (16.4)	1 (10)	0.55	4 (17.4)	0.9
Rehospitalization, n (%) (cardiovascular-caused)	14 (17.9)	5 (9.1)	1 (10)	0.48	9 (39.1)	0.002
Sepsis with worsening of heart function, n (%)	0	0	0	0	1 (3.8)	-
Poor positioning of the prosthesis/thrombosis, n (%)	2 (2.5)	1 (1.9)	0	0	1 (3.8)	0.6
Postprocedural IS or TIA, n (%)	0	0	0	0	0	-
Myocardial infarction, n (%)	0	0	0	0	0	-
Infective endocarditis, n (%)	0	0	0	0	0	-

IS=ischemic stroke; TIA=transient ischemic attack

### Independent Predictors of Early-Term Mortality

In the present study, early mortality (death in the first 30 days) occurred in 10 (12.8%) patients, who showed higher frequencies of DM, HF, chronic obstructive pulmonary disease, and NYHA classes III to IV than did survivors (*P*=0.006, *P*=0.004, *P*=0.04, and *P*=0.003, respectively). Regarding echocardiographic examinations, AVA and LVEF were lower (*P*=0.043 and *P*<0.001, respectively) and mean aortic valve gradient and pulmonary artery systolic pressure (sPAP) were higher in nonsurvivors than in survivors (*P*=0.046 and *P*<0.001, respectively). eGFR was also found to be lower in those who died (*P*<0.001). R_2_CHA_2_DS_2_-VASc, EuroSCORE II, and TAVR scores’ results were also significantly higher in nonsurvivors than in survivors (*P*<0.001, *P*<0.001, and *P*<0.001, respectively). Additionally, major bleeding requiring blood transfusion, pericardial tamponade, and acute renal failure were more frequent in patients with early mortality (*P*=0.005, *P*=0.001, and *P*<0.001, respectively).

Since both EuroSCORE II and TAVR score contain similar components and may have a negative effect on the statistical significance of each others’ results, they were not considered in the same multivariable regression model. Therefore, to determine the independent predictors of early mortality, we performed two different multivariable Cox proportional hazards regression models, with model 1 consisting of R_2_CHA_2_DS_2_-VASc and TAVR scores and model 2 consisting of R_2_CHA_2_DS_2_-VASc score and EuroSCORE II. TAVR score and EuroSCORE II were both found to be predictors of early mortality in models 1 and 2 analyses, respectively (*P*=0.01 and *P*=0.02, respectively). Of note, although there was a borderline degree of statistical significance, R_2_CHA_2_DS_2_-VASc score exhibited an association with early mortality in model 2, but not in model 1 (*P*=0.06 and *P*=0.19, respectively) (Table 4).

**Table 4:** According to ES II and TAVR scores, two different univariable and multivariable Cox proportional hazards regression analysis models for determining the predictors of the mortality at 30 days and mid-to-long-term.

Early mortality	Univariable OR (95% CI)	*P*	Model 1 Multivariable 1 OR (95% CI)	*P*	Model 2 Multivariable 2 OR (95% CI)	*P*
Acute renal failure	0.147 (0.043-0.508)	0.002	0.454 (0.101-2.033)	0.3	1.126 (0.219-5.786)	0.89
Tamponade	0.171 (0.048-0.607)	0.006	0.745 (0.073-7.637)	0.8	1.895 (0.131-27.504)	0.64
Blood transfusion	0.219 (0.063-0.756)	0.016	0.105 (0.007-1.656)	0.11	0.061 (0.002-1.545)	0.09
R_2_CHA_2_DS_2_-VASc score	1.886 (1.310-2.715)	0.001	1.407 (0.841-2.353)	0.19	1.824 (0.969-3.424)	0.06
TAVR score	1.853 (1.312-2.616)	<0.001	1.982 (1.161-3.383)	0.01	-	-
EuroSCORE II	1.718 (1.277-2.309)	<0.001	-	-	1.875 (1.100-3.198)	0.02
**Late Mortality**						
Presence of LBBB/RBBB	0.302 (0.137-0.666)	0.005	0.798 (0.315-2.017)	0.6	0.714 (0.277-1.839)	0.5
Blood transfusion	0.398 (0.171-0.923)	0.03	0.703 (0.145-3.402)	0.7	0.606 (0.107-3.441)	0.6
Tamponade	0.344 (0.129-0.921)	0.03	0.456 (0.101-2.060)	0.31	0.596 (0.116-3.067)	0.5
Acute renal failure	0.218 (0.093-0.511)	<0.001	0.648 (0.200-2.103)	0.5	0.809 (0.222-2.948)	0.7
Rehospitalization	0.243 (0.104-0.570)	0.001	0.591 (0.210-1.664)	0.3	0.505 (0.180-1.415)	0.2
R_2_CHA_2_DS_2_-VASc score	2.065 (1.599-2.667)	<0.001	1.696 (1.237-2.325)	0.001	1.670 (1.203-2.319)	0.002
TAVR score	1.519 (1.255-1910)	<0.001	1.275 (0.954-1.704)	0.1	-	-
EuroSCORE II	1.495 (1.219-1.893)	<0.001	-	-	1.396 (1.081-1.804)	0.01

CI=confidence interval; EuroSCORE II or ES II=European System for Cardiac Operative Risk Evaluation II; LBBB=left bundle branch block; OR=odds ratio; RBBB=right bundle branch block; TAVR=transcatheter aortic valve replacement

**Fig. 1:**
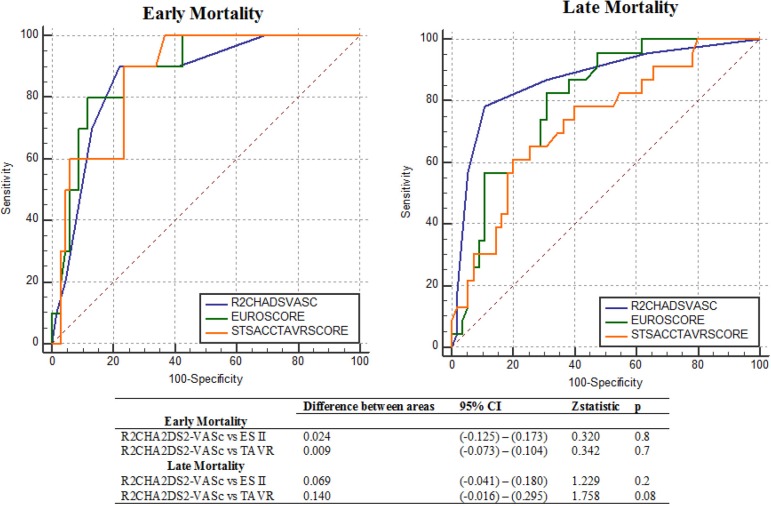
ROC curves of the R2CHA2DS2-VASc (blue), ES II (green), and TAVR scores (orange) for detecting early and mid-to-long-term mortality. ACC=American College of Cardiology; CI=confidence interval; EuroSCORE II or ES II=European System for Cardiac Operative Risk Evaluation II; ROC=receiving operating characteristic; STS=Society of Thoracic Surgeons; TAVR=transcatheter aortic valve replacement

To test the predictive performance of those scores, we performed ROC curve analysis (Table 5). The area under the curve (AUC) for 30-day mortality was 0.886 (95% CI: 0.794-0.947, *P*<0.001) with ES II, 0.871 (95 % CI: 0.775-0.936; *P*<0.001) with TAVR score, and 0.862 (95% CI: 0.765-0.929; *P*<0.001) with R_2_CHA_2_DS_2_-VASc score. A statistical comparison of ROC curve analysis outcomes revealed no significant difference between the AUC values of these scoring systems within 30 days, with *P*>0.05 (Figure 1).

**Table 5:** Predictive power of risk score modalities.

Risk scores	AUC	95% CI	Z statistic	Cuttoff value	Sens.	Spec.	*P*
**Early mortality**							
R_2_CHA_2_DS_2_-VASc	0.862	0.765-0.929	6.486	> 5	90	78	<0.001
EuroSCORE II	0.886	0.794-0.947	8.317	> 6.96	80	88	<0.001
TAVR	0.871	0.775-0.936	7.940	> 3.88	90	76	<0.001
**Late mortality**							
R_2_CHA_2_DS_2_-VASc	0.870	0.776-0.964	7.801	> 6	74	89	<0.001
EuroSCORE II	0.801	0.703-0.899	5.990	> 4.68	83	69	<0.001
TAVR	0.730	0.610-0.852	3.700	> 3.88	61	80	0.002

AUC=area under the curve; CI=confidence interval; EuroSCORE II=European System for Cardiac Operative Risk Evaluation II; Sens=sensitivity; Spec=specificity; TAVR=transcatheter aortic valve replacement

### Independent Predictors of Mid-To-Long-Term Mortality

**Fig. 2:**
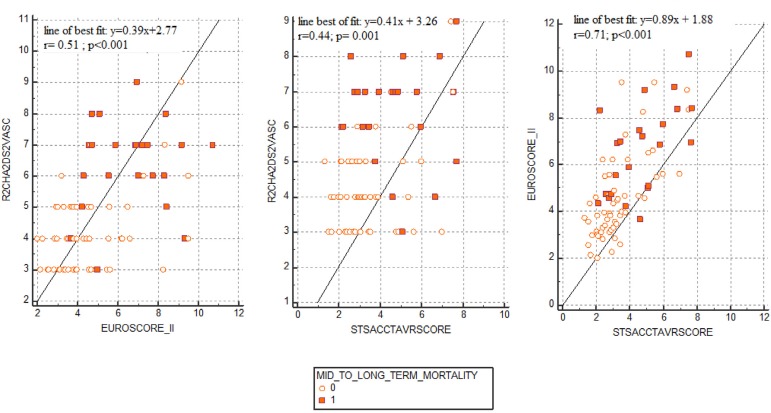
Correlation analyses of R2CHA2DS2-VASc, ES II, and TAVR score models. ACC=American College of Cardiology; EuroSCORE II or ES II=European System for Cardiac Operative Risk Evaluation II; STS=Society of Thoracic Surgeons; TAVR=transcatheter aortic valve replacement

Mid-to-long-term mortality (> 30 days) was observed in 26 (33.3%) patients with higher frequencies of DM, HF, vascular disease, history of CVA, and NYHA classes III to IV as compared with survivors (*P*=0.05, *P*=0.002, *P*=0.03, *P*=0.01, and *P*=0.001, respectively). Indications of conduction abnormalities including LBBB or RBBB were also more frequently seen on nonsurvivors’ ECGs (*P*=0.003). In echocardiographic and blood sample analyses, sPAP values were higher (*P*=0.004) and eGFR and LVEF findings were lower in nonsurvivors than in survivors (*P*<0.001 and *P*=0.004, respectively). Pericardial tamponade, acute renal failure, and cardiovascular-caused hospitalizations were more frequent in patients who died (*P*=0.03, *P*=0.003, *P*=0.002, respectively). Nonsurvivors had higher R_2_CHA_2_DS_2_-VASc, EuroSCORE II, and TAVR scores results than survivors (*P*<0.001, *P*<0.001, and *P*=0.001, respectively). An analysis of Pearson’s corelation coefficient revealed that R_2_CHA_2_DS_2_-VASc score was moderately correlated with EuroSCORE II and TAVR score (r=0.51, *P*<0.001 and r=0.44, *P*=0.001, respectively), while, as expected, both EuroSCORE II and TAVR score showed good correlation with each other (r=0.71, *P*<0.001). Scatterplot diagrams are presented in Figure 2.

**Fig. 3:**
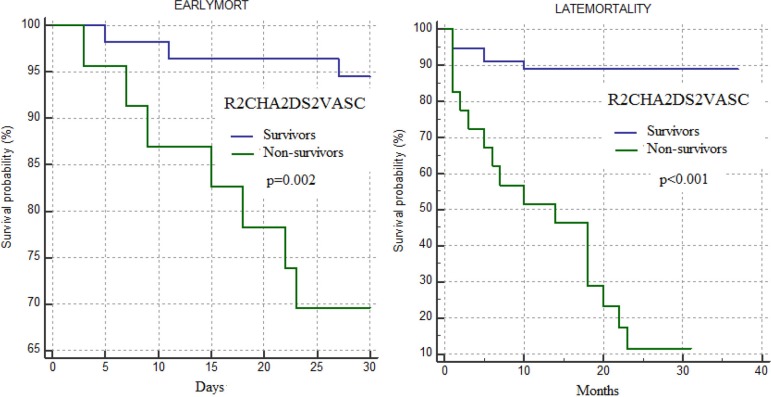
Kaplan-Meier plots of survival curves of patients with low and high R2CHA2DS2-VASc score categories. ‘0’ (blue) means low R2CHA2DS2-VASc score and ‘1’ (green) means high R2CHA2DS2-VASc score.

R_2_CHA_2_DS_2_-VASc score and EuroSCORE II were found to be statistically significant predictors of mortality in model 2 (*P*=0.002 and *P*=0.01, respectively), while only R_2_CHA_2_DS_2_-VASc score was found to be a predictor for late mortality in model 1 (Table 4). A comparison of ROC analysis findings showed that R_2_CHA_2_DS_2_-VASc score (AUC: 0.870, 95% CI: 0.776-0.964; *P*<0.001) was not significantly different from EuroSCORE II (AUC: 0.801, 95% CI: 0.703-0.899; *P*<0.001) (*P*=0.2). On the other hand, although it was not found to be statistically significant, the AUC value of R_2_CHA_2_DS_2_-VASc score was greater than the AUC value of TAVR score (AUC: 0.730, 95% CI: 0.610-852; *P*=0.002) (*P*=0.08) (Figure 1). A cutoff value of six points for R_2_CHA_2_DS_2_-VASc score showed a sensitivity of 74% and a specificity of 89% (Table 5). Kaplan-Meier curves were used to represent mortality in patients divided into low-risk (R_2_CHA_2_DS_2_-VASc score < 6 points) and high-risk (R_2_CHA_2_DS_2_-VASc score ≥ 6 points) mortality groups during the follow-up period for up to three years after TAVR (Figure 3).

## DISCUSSION

In the present study, we investigated classical risk prediction models (EuroSCORE II and TAVR score) along with a relatively new model (R_2_CHA_2_DS_2_-VASc score) so as to attempt to predict both short- and long-term outcomes. Beyond the significant utility of scores assessed in our research, our main finding is the limited accuracy of a particular score to indicate both short- and long-term outcomes. This study highlights the need for a novel score, which inevitably must combine several clinical and surgical parameters.

To the best of our knowledge, this is the first study to comprehensively consider both surgical scores and the newly defined R_2_CHA_2_DS_2_-VASc score (which includes chronic kidney disease and the presence of RBBB or LBBB in addition to traditional CHA_2_DS_2_-VASc score variables) in predicting mid-to-long-term mortality in patients who underwent TAVR. In accordance with the literature, in the present study, we found that EuroSCORE II and TAVR score were associated with early death prediction^[4,13,14]^, but R_2_CHA_2_DS_2_-VASc score was not. In the present study, patients with early mortality had higher frequencies of respiratory insufficiency (severe chronic obstructive pulmonary disease) and NYHA classes III to IV, which are the variables of EuroSCORE II and TAVR score. Additionally, pulmonary HT, a variable of EuroSCORE II, was found to be higher in nonsurvivors than in survivors. All aforementioned variables are not part of the R_2_CHA_2_DS_2_-VASc score and are proposed to be predictive of early mortality^[15,16]^. This may partially explain why R_2_CHA_2_DS_2_-VASc score did not predict early mortality in this study. On the other hand, the current study demonstrated that R_2_CHA_2_DS_2_-VASc score and EuroSCORE II were significantly associated with late mortality prediction, while TAVR score was not. Recently, Hamid et al.^[10]^ observed that a R_2_CHA_2_DS_2_-VASc score of seven points or more was strongly associated with short-term (30 days) and one-year all-cause mortality in patients who underwent TAVR. In the present study, although we did not determine a significant association between R_2_CHA_2_DS_2_-VASc score and early mortality, we did demonstrate that a R_2_CHA_2_DS_2_-VASc score of six points or more was a significant predictor of mid-to-long-term mortality (death after 30 days but before 37 months). Compatible with the findings of Hamid et al.^[10]^, we also observed that patients with higher R_2_CHA_2_DS_2_-VASc scores had a worse prognosis in the mid-to-long-term postoperative period. Although controversial data exist in the literature, some variables of the R_2_CHA_2_DS_2_-VASc score (*i.e*., DM, coronary artery disease [CAD], PAD, LVEF, RBBB on baseline ECG, HF, or lower LVEF) and of the EuroSCORE II (*i.e*., extracardiac arteriopathy, diabetes on insulin, LVEF) were proposed to be related with mid-to-long-term mortality^[17-22]^. Indeed, the meta-analysis of Sankaramangalam et al.^[19]^ and the study of Mancio et al.^[21]^ concluded that the presence of CAD was more related to all-cause mortality at one year than to either procedural-related mortality or early mortality^[19,22]^. In an analysis of the STS/ACC TVT registry, patients with PAD undergoing transfemoral TAVR showed a higher incidence of readmission and death during one year of follow-up^[23]^. In another study, DM was not found to be significantly associated with short-term mortality in TAVR, but it was significantly associated with long-term mortality^[17]^. Although in the present study cardiovascular-related rehospitalization was not an independent predictor for mid-tolong-term mortality, nonsurvivors demonstrated a higher number of readmissions. Additionally, the risk of late (> 30 days) readmission after TAVR was mainly determined by the presence of chronic HF, peripheral vascular disease, and chronic kidney disease, which have also been systematically found to be strong risk factors for mortality in TAVR^[24-26]^.

In the light of the existing literature, the aforementioned reasons may explain about why R_2_CHA_2_DS_2_-VASc score and EuroSCORE II predicted the mid-to-long-term mortality, while TAVR score, which does not include these aforementioned variables, did not. Besides, the STS/ACC TAVR score was developed to estimate in-hospital mortality rather than late mortality^[4]^.

Despite the fact that the findings of our study are not generalizable to every patient, we can still draw some key conclusions, as follows: first, TAVR score and EuroSCORE II predicted early mortality (≤ 30 days), but R_2_CHA_2_DS_2_-VASc score did not; second, R_2_CHA_2_DS_2_-VASc score and EuroSCORE II were statistically significant predictors for mid-to-long-term mortality in patients who underwent TAVR, but the STS/ACC TAVR score was not; and, third, patients with R_2_CHA_2_DS_2_-VASc score of six points or more were found to be at high risk of death for up to 37 months postoperation.

### Study Limitations

Our study has some limitations that should be noted. First, our study was a retrospective analysis of a single-center registry including a small number of consecutive patients who underwent TAVR. Second, the present study was designed to test the relationship between a risk score designed to predict the risk of stroke and the TAVR procedure-related mortality.

## CONCLUSION

In conclusion, R_2_CHA_2_DS_2_-VASc score may be considered as a handy risk stratification tool that could assist clinicians in their decision-making and may advise individual patients of their midto-long-term risk due to its ability to predict late mortality in patients undergoing TAVR.

**Table:** 

Authors' roles & responsibilities	
MS	Substantial contributions to the conception or design of the work; or the acquisition, analysis, or interpretation of data for the work; drafting the work or revising it critically for important intellectual content; final approval of the version to be published
SO	Substantial contributions to the conception or design of the work; or the acquisition, analysis, or interpretation of data for the work; final approval of the version to be published
